# Shear bond strength in two types of indirect orthodontic cementation

**DOI:** 10.4317/jced.61800

**Published:** 2024-08-01

**Authors:** Arnaldo-Alfredo Munive-Mendez, Rafael Morales-Vadillo, Janet-Ofelia Guevara-Canales

**Affiliations:** 1 Faculty of Dentistry, “Universidad de San Martín de Porres”, Lima - Peru

## Abstract

**Background:**

To compare the shear bond strength of brackets cemented to dental enamel according to the cementation techniques.

**Material and Methods:**

Experimental study. We used 90 premolars and placed them in printed polylactic acid (PLA) filament models to simulate the dental arch shape and to then cement brackets using the direct, indirect technique with Transbond™XT and indirect technique with Orthocem®. Then, we carried out a shear bond strength test using a universal testing machine, and we evaluated the enamel surface using the adhesive resin remaining index. Dunn’s test was used for the inferential statistical analysis of shear bond strength, and Fisher’s exact test was used for the adhesive resin remaining index.

**Results:**

The shear bond strength of the brackets recorded mean values of 16.74±4.48Mpa, 15.93±6.49Mpa and 12.09±5.07Mpa in the direct, indirect technique with Transbond™XT and indirect technique with Orthocem® respectively. At an inferential level, a lower statistically significant difference was found in the indirect group with OrthoCem® in contrast to the other two groups. In the evaluation of resin remaining after detachment, the direct technique group registered 46.7% of teeth with more than half of resin remaining and the indirect technique groups with Transbond™XT and Orthocem® registered less than half of resin remaining with an incidence of 53.3% and 43.3% respectively. At an inferential level, a statistically significant difference between groups was evidenced.

**Conclusions:**

The indirect cementation technique using Transbond™ XT is more recommended since it presents a higher shear bond strength than using Orthocem®.

** Key words:**Orthodontics, Adhesion, orthodontic adhesives, shear bond strength.

## Introduction

Orthodontics and maxillary orthopedics is a dental specialty that aims to correct dental and skeletal alterations of the maxillomandibular complex (1). Treatments related to this specialty aim to correct occlusion, restore masticatory function and improve dental and facial esthetics, favoring the patient’s self-esteem and improving the patient’s quality of life (2).

One of the factors enabling these objectives to be achieved is the correct positioning of the fixed orthodontic appliances, composed of brackets and orthodontic tubes (3). In 1972, Silverman *et al*. (4) proposed the technique of indirect cementation, considering it to be more advantageous than direct cementation.

This technique consists of positioning the brackets on a model and performing the transfer directly in the mouth with the help of a splint. Over the years, this technique has undergone modifications and tests, achieving greater accuracy and reduced clinical care time (5).

Considering that appliance installation is the most time-consuming procedure in orthodontics, the indirect cementation technique helps in its optimization. In addition, as the use of the indirect technique results in less clinical work time it also reduces saliva contamination (6).

However, it is necessary to make a continuous evaluation and adaptation of indirect cementation procedures, for an increasingly accurate, safe, accessible and replicable work in our country. To this end, it is necessary to evaluate parameters such as shear bond strength and the amount of remaining resin (7).

Given the above, the objective of our study is to compare the shear bond strength of brackets cemented to dental enamel according to the cementation technique.

## Material and Methods

This was an experimental, cross-sectional, *in vitro* study, which received ethics approval from the ethics committee board of the Faculty of Dentistry of the San Martín de Porres University. (Resolution: ACTA N°004-2020-CEI/FO-USMP).

Ninety premolars were obtained from patients treated in private dental clinics. These premolars were then distributed in three groups according to the fixed orthodontic appliance cementation technique used:

- Group 1: 30 premolar teeth bonded to brackets using the direct technique (control group).

- Group 2: 30 premolar teeth bonded to brackets using the indirect technique with hot melt silicone splint using Transbond™ XT cement.

- Group 3: 30 premolar teeth bonded to brackets using the indirect technique with hot melt silicone splint using OrthoCem® cement.

We selected premolar teeth that were extracted from patients due to orthodontic reasons. Exclusion criteria were teeth with shape anomalies, enamel defects, extensive carious lesions, and prosthetic treatment. We also used clinical records to assess if the patient was a smoker or had previous tooth whitening treatment since both factors can influence shear bond strength (8,9).

The private clinics that donated teeth were instructed that after the extraction, the teeth should be disinfected with a 0.5% chlorine solution and stored in plastic containers with 0.9% physiological saline solution at room temperature. Teeth were kept in these conditions until they were collected.

Subsequently, the computer-aided design software SolidWorks™ 2019 (SolidWorks Corp., Massachusetts, USA) was used to design a PLA mock-up to position the teeth in groups of 16 in the shape of a dental arch. Additionally, attachments were designed to simulate a dental arch impression and facilitate the positioning of the individual pieces in the universal testing machine. This model has been published as a utility model application at the National Institute for the Defense of Competition and Protection of Intellectual Property of Peru (application number PE20220641Z).

The designs were produced on the Inventor series 3D printer (Flashforge 3D Technology Co., Zhejiang, China) using polylactic acid or PLA+ filaments (SUNLU Industrial CO., Zhuhai, China) of 1.75mm calibre.

We proceeded to fix the teeth using fast-curing acrylic to the mock-up printed in PLA+ and assembled them by arches, obtaining a total of 6 arches of 15 teeth each to reach the required sample size.

Then, the brackets were cemented according to the type of cementation technique:

-Direct technique with Transbond™ 

For the 30-teeth group with the Transbond™ XT direct technique, the buccal surface was etched for 15 seconds with 37% orthophosphoric acid gel. Then, the surface was washed with water using the three-way syringe for 20 seconds and dried with compressed air for 10 seconds. Next, the adhesive conditioner (Transbond™ XT primer, 3M Unitek, Monrovia, CA, USA) was applied. We then placed the Transbond™ XT cement at the base of the bracket and positioned the bracket and pressed it on the enamel surface. The excess was removed, and we applied polymerizing light for 10 seconds.

-Indirect technique

An impression was taken using Tropicalgin alginate (Zhermack SpA, Badia Polesine, Italy) of the group of teeth arranged in the shape of a dental arch. Then the cast was made using Pentadur type III stone plaster (Penta Industrias SAC, Lima, Peru). Consequently, a layer of acrylic isolator was applied, and we positioned the brackets on the plaster model and used Transbond™ XT or OrthoCem® cement (FGM Dental Group, Joinville, Brazil) according to the assigned group.

Subsequently, a layer of spray silicone was applied, and we made the splint using a hot melt glue gun. The application of the silicone was direct, and we verified that the extension was sufficient to cover at least 50% of the buccal surface and at least 50% of the occlusal surface.

We then proceeded to separate the plaster model from the splint. For this, the model was immersed in water at 10°C for 10 minutes, and we made sure that the brackets remained in the splint. The bracket bases were cleaned using a 110-micron aluminum oxide dental sandblaster.

The teeth were etched for 15 seconds with the orthophosphoric acid gel, then the surface was washed with water using the three-way syringe for 20 seconds, and the surface was dried with compressed air for 10 seconds.

For the group of 30 teeth with Transbond™ XT, a layer of adhesive conditioner was applied first. Then, Transbond™ XT cement was placed on the bracket base, and the splint was immediately placed on the tooth model, to which we light-cured each tooth for 20 seconds per side (10).

For the group of 30 teeth with OrthoCem®, the cement was applied on the base of the brackets, and immediately the splint was placed on the models of the teeth, and each tooth was light-cured for 20 seconds per side.

For all groups, the LED B Woodpecker 1000mW/cm2 Woodpecker LED B wireless curing light (Woodpecker Medical Instrument Co., Guilin, China) was used.

At the end of light curing, we soaked the model in water at 40ºC for 3 minutes and removed the splints.

- Evaluation of shear bond strength

The acrylic blocks were removed from the models and were adapted to be used in the universal testing machine (Instron model 3366) at a load cell of 500 N and a speed of 0.5 mm/min.

**Figure 1:**
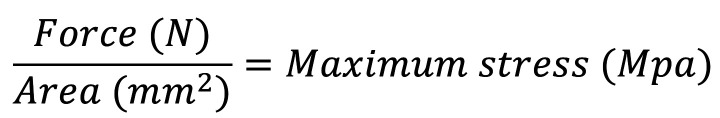
Maximum stress calculation formula.

The maximum stress in megapascals (MPa) was obtained with the following formula: (Fig. 1).

Where:

• Force is measured in Newtons and recorded by the universal testing machine.

• Area is the average square millimeters obtained by measuring the base of the bracket.

• Maximum stress is measured in megapascals, used to measure the shear bond strength.

-Assessment of the adhesive resin remaining index (ARI)

After the shear bond strength test was performed, the buccal faces were left without the bracket attached. This enamel surface was observed under the 10x binocular optical microscope, the photographic record was taken and the analysis was performed according to the ARI index.

**Figure 2:**
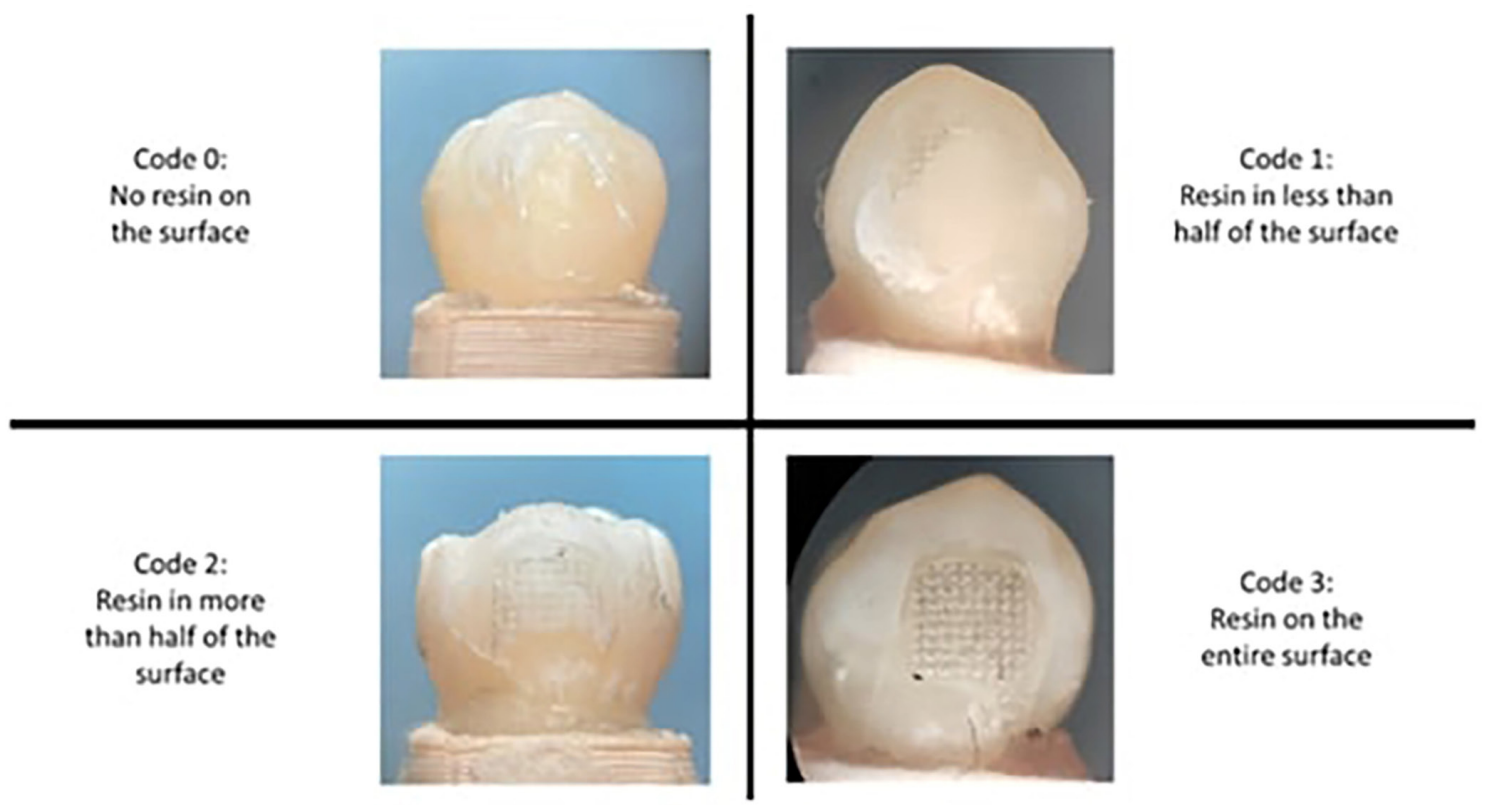
Adhesive resin remaining criteria.

In the present investigation, the four-scale modification introduced by Årtun and Bergland (11) and modified by Iglesias *et al*. (12) was used (Fig. 2).

**Figure 3:**
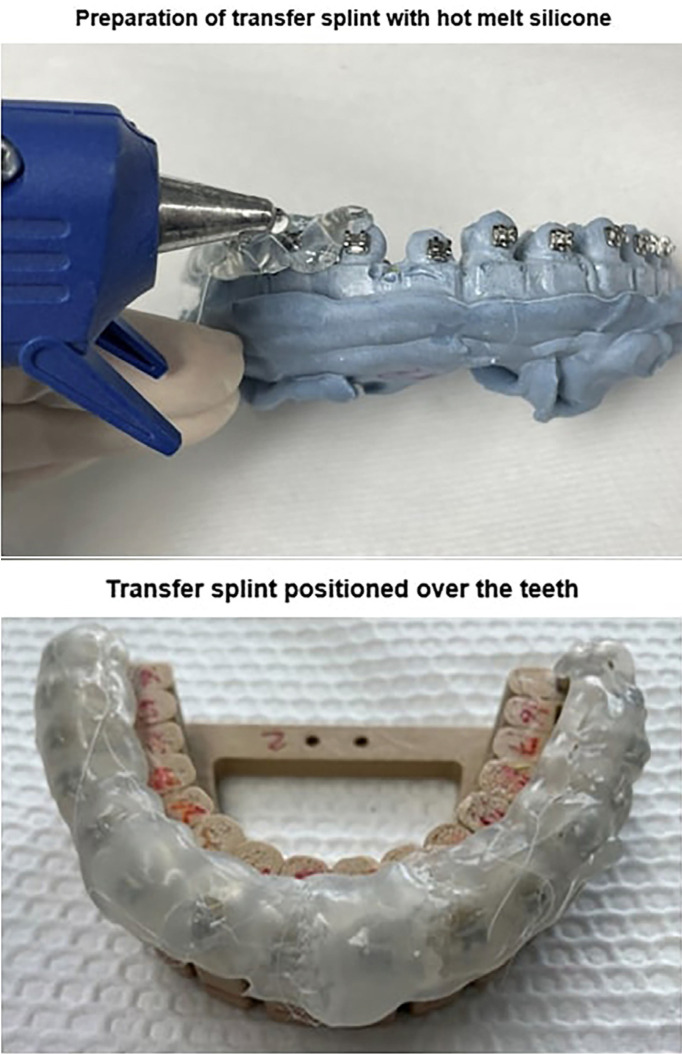
Working sequence for indirect ceentation.

A photographic record of the execution sequence was taken (Fig. 3).

-Statical Analysis

First, we obtained summary statistics (central tendency) and dispersion by group. In our inferential analysis, we used the Kruskal-Wallis test and Dunn’s post hoc test to compare shear bond strength, and Fisher’s exact test to assess the adhesive resin remaining index.

## Results

**Figure 4:**
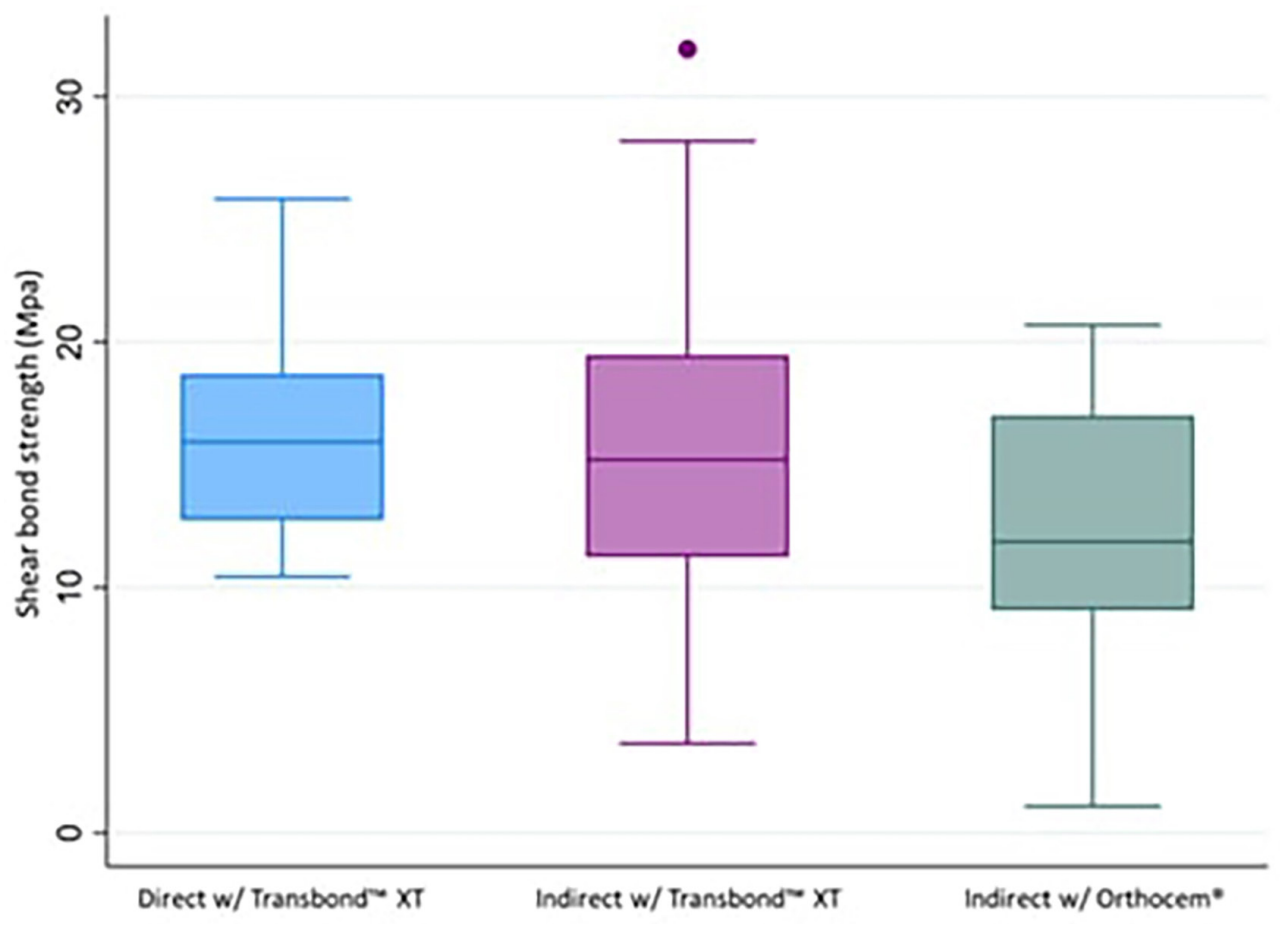
Evaluation of the shear bond strength of brackets cemented to tooth enamel using direct, indirect with Transbond TM XT and indirect with OrthoCem®.

On reviewing the descriptive statistics of the evaluation of the shear bong strength of brackets cemented to dental enamel, it can be observed that the samples using direct cementation technique with Transbond™ XT obtained a mean of 16.74±4. 48 Mpa, followed by the samples that used the indirect cementation technique with Transbond™ XT with a mean of 15.93±6.49 Mpa. On the other hand, the samples using the indirect cementation technique with Orthocem® obtained a mean of 12.09±5.07 Mpa (Fig. 4,  Table 1).

The descriptive analysis of the evaluation of the index of resin remaining after debonding of brackets cemented to the dental enamel shows that the samples worked with the direct cementation technique with Transbond™ XT obtained 0% for grade 0, 16.7% for grade 1, 46.7% for grade 2 and 36.7% for grade 3. In the group of samples which used the indirect cementation technique with Transbond™ XT, they obtained 26.7% for grade 0, 55.3% for grade 1, 16.7% for grade 2 and 3.3% for grade 3. In the group of samples using the indirect cementation technique with Orthocem®, they obtained 26.7% for grade 0, 43.3% for grade 1, 26.7% for grade 2 and 3.3% for grade 3.

**Figure 5:**
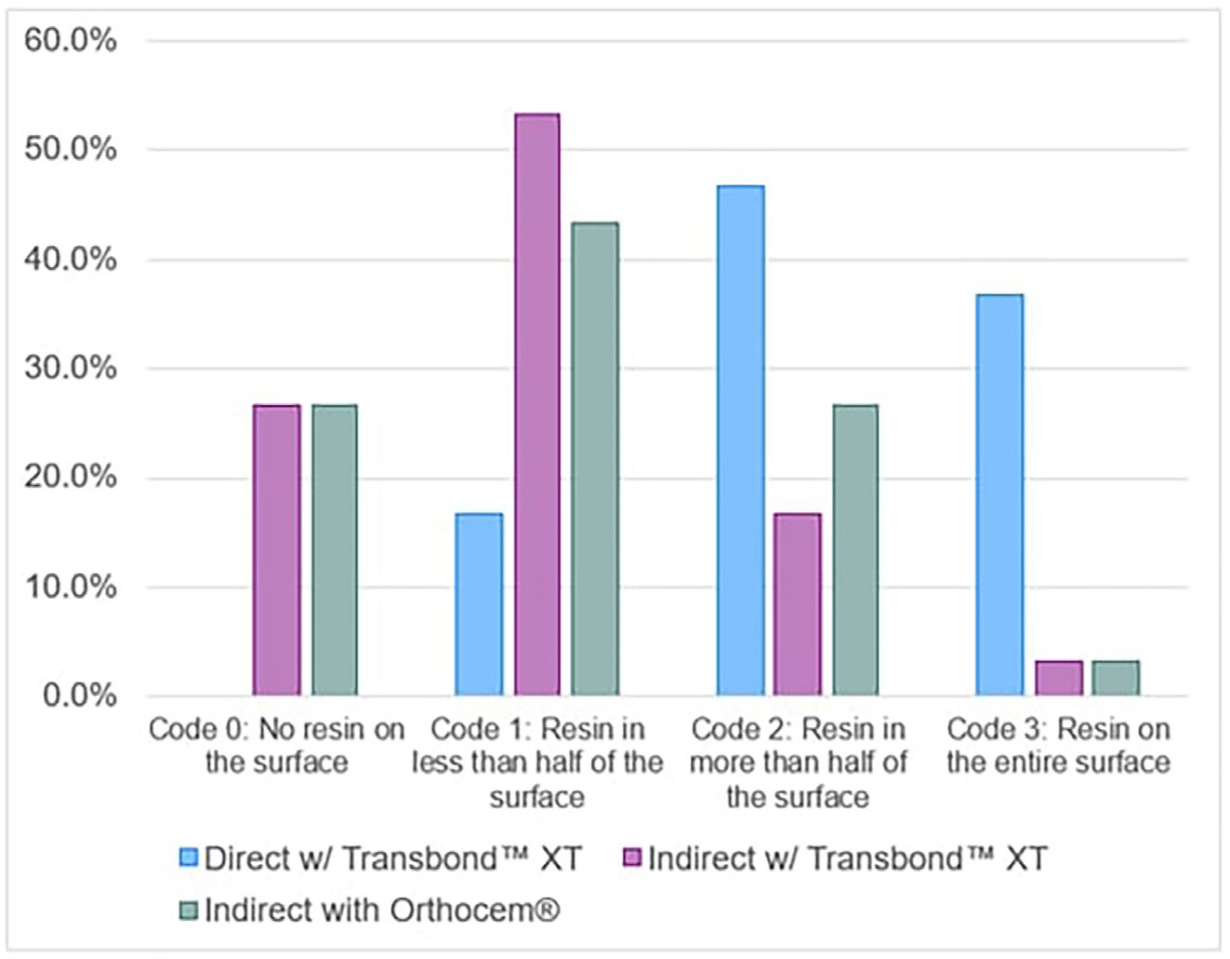
Evaluation of the rate of resin remaining after deboonding in brackets cemented to tooth enamel using the direct technique, indirect with TransbondTM XT and indirect with OrthoCem®.

In the inferential analysis, we found a *p-value*<0.05 when performing Fisher’s exact test. As such, the null hypothesis was rejected, confirming that there are significant differences between the values recorded (Fig. 5,  Table 2).

We also used the Kruskal-Wallis test to compare the shear bong strength of brackets cemented to dental enamel between cementation techniques, where we obtained a *p-value*< 0.05. Therefore, the null hypothesis was rejected, confirming that there are statistically significant differences between groups. We then performed a post hoc analysis using Dunn’s test, which showed that there is no difference between the group of samples with the direct and indirect technique with Transbond™ XT, but there are significant differences between both groups in contrast to the group of samples which used the indirect technique with OrthoCem® ( Table 3).

## Discussion

The purpose of our study was to compare the shear bond strength of brackets cemented to dental enamel according to the cementation technique.

According to Pamukçu and Özsoy (7), the indirect bonding technique of orthodontic appliances offers advantages such as shorter chair time, better control of the cement thickness between the enamel surface and the bracket mesh, and easy adjustment of the overcorrection. At the clinical level, there may be doubts about the position placed in the model versus the final result in the transfer tray. However, in the study by Castilla *et al*. (3), it is possible to confirm that there is a statistically significant precision during this process, considering that good stability and rigidity must be guaranteed in the type of transfer tray.

In terms of the type of indirect technique for bracket cementation, our study was based on the protocol proposed by Pamukçu and Özsoy (7) where they use micro-sandblasting with aluminum oxide to clean the bracket meshes before taking the transfer tray to the mouth and the protocol proposed by Munive and Caro (10) who use a transfer tray made of thermofusible silicone. The advantage of the latter modification is that it is more accessible, which allows its use to become widespread. Yuzbasioglu and Alkan (13) recommend that the transfer tray should be thin enough to facilitate the passage of the light curing light but sufficiently resistant so as not to lose precision (5).

Regarding the teeth sample selection, teeth with lesions due to fluorosis were not selected. In the study by Basalamah *et al*. (14) a similar sample of 90 premolar teeth was used, and a median shear bond strength of 8.14Mpa versus 6.57Mpa was recorded in the groups of healthy teeth and teeth with fluorosis lesions, respectively, with a significant difference between the two groups. We postulate that teeth with fluorosis present enamel with an abnormal or disorganized inorganic structure that alters adhesive efficiency (15).

We decided to have non-smoking tooth donor patients because smoking exposure can affect bracket adhesion. In the in-vitro investigation by Omar *et al*., the teeth simulated tobacco exposure presented a strength of 2.8±0.7Mpa, presenting values well below their control group (mean of 15.7±9.5Mpa) and the values recorded in our study. This decrease in shear bond strength is due to the chemical components of the cigar, which presumably interfere with the resin and the enamel interface (8).

Teeth from patients with previous tooth whitening treatment were not considered, since it can also alter the adhesion to the enamel. In Perciano *et al*., (16) 60 monocrystalline and polycrystalline brackets were cemented to bovine incisors and the shear bond strength was significantly higher after the tooth whitening sessions. Initially, the values obtained for monocrystalline and polycrystalline brackets were 49.79±12.90 and 14.35±7.80, respectively. At the end of the session, values were 54.16±10.01 and 26.44±12.40. In terms of the resin remaining index before tooth whitening, all the samples presented a code 3. As the tooth whitening sessions were carried out, they did not generate differences in the amount of resin remaining after debonding. This may be explained by the whitening sessions, which promote irregularities in the enamel and increase the mechanical retention of the orthodontic cement (17). However, Zarif *et al*. (9) reported a decrease in adhesive strength supported by an affectation of the adhesive interface between the brackets and the enamel.

It can be seen that the adhesive strength in esthetic brackets (16,18) is greater in comparison with the metal brackets used in this study. In the study by Iglesias *et al*., the shear bond strength and adhesive resin remaining index was evaluated using similar parameters to the present study. Transbond™ XT was used to obtain for the direct technique a mean of 13.50±4.00Mpa and an indirect of 11.10±3.9Mpa, presenting a similar numerical trend to the present study. The self-adhesive resinous bracket cement GC Ortho Connect™ (GC America, Alsip, Illinois, USA) was also used, obtaining a shear bond strength of 10.50±3.40Mpa compared to 12.09±5.07Mpa obtained in our study using OrthoCem®, whose cement presents similar characteristics to the one mentioned. They also found no differences between the direct and indirect cementation technique groups (12).

In the study by Shimizu *et al*., (18) a test with similar characteristics was performed, where, 8.1±1.7Mpa was obtained for the direct technique, indirect with the Sondhi™ indirect bonding kit and indirect with Transbond™ XT respectively. Upon inferential analysis, the study concluded that the three techniques evaluated had similar adhesive effectiveness. Similarly, the research by Queíroz *et al*. (19) found no difference between the direct and indirect techniques of orthodontic appliance bonding. These findings are shared by the present study when evaluating the direct and indirect techniques using Transbond™ XT.

Regarding the results on the adhesive resin remaining index, Iglesias *et al*. (12) found in the direct cementation group with Transbond™ XT a higher incidence in code 1 (33.3%), while in the present study, a higher incidence was recorded for code 2 (46.7%). These values register an effective bond in the enamel, considering that a higher incidence in lower codes may be associated with a greater possibility of adhesive failure and imminent detachment of the orthodontic appliance, while in higher codes it implies greater damage to the enamel during the excess resin removal and polishing procedure (14).

It is possible to make modifications to the bonding technique given the slight difference in shear bond strength in the indirect technique using Orthocem® (12.09±5.07Mpa) versus the same technique using Transbond™ XT (15.93±6.49). Fonseca-Silva *et al*. performed tests on brackets of similar brands (Morelli® Sorocaba, São Paulo) and similar characteristics (conventionally bonded metal) and obtained average values of 7.8±3.6Mpa and 11.3±2.7Mpa for the groups where Orthocem® and Orthocem® were used, adding the universal adhesive Ambar® (FGM Dental Group, Joinville, Brazil), which upon statistical analysis showed that there was no significant difference between the two protocols (20).

When performing in-vitro studies where the teeth must be placed together as a dental arch to simulate procedures involving dental impressions, the difficulty lies in transferring them in a unitary form to the universal testing machine or equivalent. Especially when it is desired to perform experimental designs of the longitudinal type where multiple procedures are desired to be performed as an arch and maintain the same joint position of the dental pieces. In the present study, we proposed to use a movable block system with a space to fix the root portion of an uniradicular tooth with fast-curing acrylic. Yi *et al*. (21) fabricated an acrylic hemiarch manually, which adds one more step to perform individual tooth separation. Shimizu *et al*. (18) used individual acrylic models. However, it does not allow them to interact with the teeth as an arch.

For future studies, color changes could be evaluated by immersing the samples in dyed solutions (e.g., coffee). Similarly, enamel roughness could be evaluated using an optical roughness meter employing the interferometric technique at 20x magnification (19).

It is important to consider replicating the characteristics of the study in patients with orthodontic appliances. However, it is necessary to carry out *in vitro* studies to guarantee optimum adhesive effectiveness, so that patients are not harmed by the delay in treatment due to bracket detachment.

It would be ideal to consider evaluating the adhesive effectiveness in each type of tooth because each one presents variable morphologies that could influence the study results. However, the collection of different types of teeth for the study would be unfeasible in comparison with premolars since these can be collected from patients requiring extractions due to tooth discrepancies in orthodontic treatment.

## Conclusions

There is no statistically significant difference between using the direct and indirect cementation technique with Transbond™ XT while the indirect technique with OrthoCem® obtains significantly lower shear bond strength values and adhesive resin remaining index.

## Figures and Tables

**Table 1:** Evaluation of the shear bond strength of brackets cemented to tooth enamel using direct, indirect with Transbond™ XT and indirect with OrthoCem®.

Type of cementation	Shearing strength (Mpa)			
	Mean ± sd	Median	Min	Max
Direct with Transbond™ XT	16.74±4.48	15.93	10.45	25.83
Indirect with Transbond™ XT	15.93±6.49	15.20	3.64	31.93
Indirect with Orthocem®	12.01±5.07	11.87	1.08	20.70

Statistical significance (*p*<0.05)

**Table 2:** Comparison of the rate of resin remaining after debonding in brackets cemented to tooth enamel using the direct technique, indirect with Transbond™ XT and indirect with OrthoCem®.

Type of cementation	Adhesive remnant index (ARI)				
	Code 0: No resin on the surface	Code 1: Resin in less than half of the surface	Code 2: Resin in more than half of the surface	Code 3: Resin on the entire surface	p*
Direct with Transbond™ XT	0 (0%)	5 (16.7%)	14 (46.7%)	11 (36.7%)	<0.001
Indirect with Transbond™ XT	8 (26.7%)	16 (53.3%)	5 (16.7%)	1 (3.3%)	
Indirect with Orthocem®	8 (26.7%)	13 (43.3%)	8 (26.7%)	1 (3.3%)	

* Fisher test Statistical significance (*p*<0.05)

**Table 3:** Comparison of shear bond strength of brackets cemented to tooth enamel using direct, indirect with Transbond™ XT and indirect with OrthoCem®.

Type of cementation	Shearing strength (Mpa)	
	Mean ± sd	p*
Direct with Transbond™ XT	16.74±4.48^a^	0.006
Indirect with Transbond™ XT	15.93±6.49^a^	
Indirect with Orthocem®	12.09±5.07^b^	

* Kruskal-Wallis test Dunn test: Dissimilar letters indicate statistical significant difference Statistical significance (*p*<0.05)

## Data Availability

The datasets used and/or analyzed during the current study are available from the corresponding author.
